# Teeth of the Lower Jaw at Birth

**Published:** 1893-10

**Authors:** Frank Abbott

**Affiliations:** New York


### International Dental Journal.

Vol. XIV. October, 1893. No. 10.

### Original Communications.[1]

### TEETH OF THE LOWER JAW AT BIRTH.[2]

BY DR. FRANK ABBOTT, NEW YORK.

Heitzmann and Bödecker (*Independent Prac.*, vols. vii.–ix.) in their "Contributions to the History of the Development of Teeth" brought their researches up to the ninth month of fœtal life. I have followed these studies up to the time of birth, in order to ascertain at this period the progress of development of the temporary as well as the permanent teeth during the last months of fœtal life. Two lower jaws of apparently well-developed new-born babes were excised soon after death, stripped of their soft tissue, and placed for preservation in alcohol. Afterwards they were placed in a one-half-of-one-per-cent. solution of chromic acid for the purpose of decalcifying the hard tissues, and at the same time to preserve the soft structures. I call attention especially to this method, since it has proved in our hands to be the safest for the preservation of the teeth.

Previous descriptions of developing teeth have been questioned in Germany, on the ground that the preservation of the tissues was

[1] The editor and publishers are not responsible for the views of authors of papers published in this department, nor for any claim to novelty, or otherwise, that may be made by them. No papers will be received for this department that have appeared in any other journal published in the country.

[2] Abstract from a paper read before the World's Columbian Dental Congress, Chicago, 1893.

not thoroughly done. After repeated renewals of the chromic-acid solution, the jaws were supposed to have become soft enough to be cut with a razor; but it was found that the central portions after they had been cut up into blocks were still hard, and had to be again immersed in the chromic-acid solution for the completion of the decalcification. The blocks had been obtained from the right half of each of the jaws, and were cut radiatingly, in order to obtain antero-posterior, vertical sections. The blocks were embedded in celloidin, sliced into thin sections, and each section numbered with the utmost care, in order to keep the succession of the teeth unbroken. Thus the section could be examined and those selected for mounting which contained teeth, or features belonging to the process of the development of teeth. The sum total of the sections thus obtained was one hundred and twenty-five. Out of these, again, only those were selected for study and drawing which showed the teeth in the greatest perfection, the most central section being selected for drawing.

Before entering into the description of the teeth I wish to say that one of the jaw-bones, under the microscope, was found to be slightly rachitic, as proved by some scanty islands of hyaline cartilage found in the bone-tissue; though the baby was to all appearances normal. Previous observations have established the fact that congenital rachitis is first shown under the microscope by a retardation of development of bone-tissue in the lower jaw, before any abnormal systems appear to the naked eye, either on the skull or shaft-bones. It is evident that in this case the rachitic process has caused a slight delay in the formation of dentine and enamel. This assertion is clearly established by a comparison with the sections from the second jaw, in which there was not the slightest symptom of rachitis. The best specimens, however, and most of the drawings, were obtained from the first jaw. The slight deficiency in the deposition of lime-salts enabled me to obtain nearly perfect sections, whereas nearly all of the sections of the second jaw were very imperfect.

Another difficulty arose; as before stated, all the sections were made in an antero-posterior vertical direction. Some of the temporary and most of the permanent front teeth are, as is well known, irregular, or devious within the jaw, so much so that the sections do not always fully comply with the expression; consequently some of the drawings may not correspond to the greatest height or designed diameter of the tooth illustrated.

The first tooth met with was, of course, the *central incisor*. The papilla of this tooth exhibited a bluntly ovoid shape, with a somewhat broader top, and slightly tapering to the lower end. As a matter of course, the whole papilla, at this stage, is destined for the production of the crown of the tooth only, and there is no trace yet present of the future root.

The papilla is composed of a myxo-fibrous connective tissue throughout its main bulk, and is scantily supplied with capillary blood-vessels. The periphery of the papilla shows a row of odontoblasts, only at the labial aspect, and not entirely here, even, since the lowest third of the papilla lacks in this respect. Here, and on half of the summit, as well as along the whole lingual aspect, the surface of the papilla exhibits a myxomatous medullary tissue, without an admixture of delicate bundles of fibrous connective tissue, with which the rest of the papilla is abundantly supplied. The summit of the papilla is coated with a dentine-cap and an enamel-cap, the former being in its diameter slightly in excess of that of the latter. The dentine-cap is further advanced in development on the labial than on the lingual aspect; it exhibits a non-calcified portion nearest the papilla, which has assumed a deep stain from carmine; whereas the calcified portion remained unstained.

The border-line between the two is marked by the well-known globular deposit of lime-salts. The enamel-cap, of a greenish-brown color (due to the chromic acid), stops short of the dentine-cap, and is, at its peripheral portion, made up of regularly-developed prisms. The inner epithelium, from which arise the ameloblasts, produces a perfect row all around the papilla with the exception of the apex. The place of recurvation to the outer epithelium, which latter is considerably broken at this stage of development, is noticeably deeper on the labial than on the lingual aspect. The space between the inner epithelium and the already-formed tooth is produced by a detachment of the former from the latter, and plainly shows a layer of protoplasmic bodies into which the ameloblasts have retrogressed before becoming infiltered with lime-salts. Since this feature is pronounced in all the teeth of the jaws under consideration, the specimens are of great value in assisting at least in settling the still mooted question as to the mode of development of enamel. I propose to dwell more fully upon this topic after the description of the cuspid.

The central permanent incisor is a formation with a considerably broader papilla, but less-developed enamel- and dentine-caps than the corresponding temporary tooth. The papilla is made up exclusively of medullary tissue supplied with scanty blood-vessels, none of which could be seen clearly in the specimen from which the illustration was taken. Both the inner and the outer epithelium were plainly visible. The enamel organ is much less advanced in the formation of myxomatous tissue, especially at the point of recurvation of the epithelia, than in the temporary tooth.

The lateral temporary incisor has a papilla much longer than that of the central; at the same time it is of a more cylindrical form, otherwise of a structure identical in all respects. On the top of the papilla we notice a rounding labial, and a sharply-pronounced angle at the lingual portion. We observe a row of odontoblasts only at the central portion of the labial surface, at the summit, and the upper two-thirds of the lingual aspect, while the rest of the surface is occupied by a medullary tissue destitute of fibrous elements, the same as in the central incisor. The dentine-cap is considerably broader than that of the central incisor, and extends farther down upon the labial surface. It, of course, forms a decided angle at the lingual portion of the top of the tooth. The border-line between the non-calcified and calcified portions of the dentine is more conspicuous by the presence of globular deposits of lime-salts than in the central incisor. The enamel-cap is of about the breadth of that of the central, and strictly follows, in its general contour, the dentine-cap. A pronounced feature in this tooth is the medullary tissue occupying the space between the row of ameloblasts and the surface of the tooth. The myxomatous enamel-organ in both temporary incisors does not show a well-developed reticulum, but in its stead a finely granular protoplasmic mass. Obviously a stage of development of the myxomatous reticulum is progressive formation.

The lateral permanent incisor has an oblong papilla, notable for the abrupt stopping of the enamel- and dentine-caps at the lingual aspect, and also for the precipitous lingual portion of its upper half. The blood-vessels are comparatively few in number, traversing the medullary tissue of the papilla. A fully-developed row of odontoblasts is to be seen only at the lingual and posterior cutting-edge; the rest of the cutting-edge is mostly destitute of these bodies. The rest of the papillary surface shows only rows of medullary corpuscles tending towards the formation of odontoblasts. A conspicuous feature in this specimen is the difference between the points of recurvation of the epithelia, which at the labial aspect embraces quite a portion of the bottom of the papilla; while at the lingual it barely reaches down one-half its length.

The temporary cuspid has a papilla which, it will be observed, is somewhat triangular in shape, with a sharply-pointed apex, and irregularly rounded at the base, with the lingual portion extending down slightly beyond the labia. The structure of the papilla does not materially differ from that of the incisors, the amount of capillaries also being about the same. A row of odontoblasts is observable only at the lower portions of the surface of the tooth, on the labial and lingual aspects. The summit is occupied by a well-pronounced myxomatous, the rest of the surface by a medullary tissue. The dentine-cap is very broad and sharply pointed at its summit. It extends far down along the surface of the papilla, more so anteriorly than posteriorly. The non-calcified position is decidedly narrower, and the globular boundary-line less pronounced than in the incisors. The enamel-cap is likewise very broad, stopping short of the dentine-cap, and is regularly developed in every respect.

The sections obtained from the cuspids were so perfect that they could be utilized advantageously to assist in settling certain moot questions as to the development of dentine and enamel. In the first place, the odontoblasts have, since their discovery, been considered by the majority of histologists as the dentine-formers proper.

It was Heitzmann and Bödecker, in their above-quoted article, who first denied the direct transformation of odontoblasts into dentine, but claimed that they are first broken up into medullary corpuscles, at their distal ends, which become infiltrated first with a glue-yielding basis-substance, and afterwards with lime-salts, and that the offshoots of the original odontoblasts, being the dentinal fibrillæ, ran between the calcified medullary corpuscles, in their respective canaliculi. Every odontoblast sends off one or more fibrillæ. This fact, with some observers, renders the formation of the basis-substance not clearly understood. So great, indeed, has been the difficulty that E. Klein, of London, and R. R. Andrews, of Cambridge, have resorted to specific fibre-cells for the production of these fibrillæ, whilst the odontoblasts proper produce the basis substance.

This view was met at the time by a demonstration, showing that the "fibre cells" were wedge-shaped odontoblasts, and most numerous where the odontoblasts are arranged in a sharply-curved line, especially at the summit of the papilla. In the pig's fœtus, for instance, the summit of the papilla is occupied almost exclusively by such narrow and wedge-shaped odontoblasts. The difficulty is, however, easily overcome by the demonstration of medullary corpuscles at the periphery of the papilla, directly beneath the already-formed dentine. In my specimens, especially the cuspid, one is struck by the scantiness of rows of odontoblasts and the presence of medullary elements in their stead; one row being visible on a portion of the labial, another on a portion of the lingual surfaces only. Not infrequently the odontoblasts are arranged at acute angles to the dentinal canaliculi. This feature possibly may be attributed to the hardening process, and the subsequent disfigurement by shrinkage, although good reasons may be adduced to the contrary, as the specimens are so perfect in all other respects. The greater area of surface of the papilla is occupied by a medullary tissue, claimed to be the dentine-former proper. In our cuspid the summit is occupied by a myxomatous tissue, approaching in gracefulness almost that of the enamel-organ. Between this myxomatous tissue and the border of the non-calcified dentine clusters of indifferent or medullary corpuscles are seen, arranged longitudinally along the surface of the papilla, therefore not as yet arranged for the formation of dentine. Beneath the myxomatous tissue we find vascularized myxo-fibrous tissue, constituting the main bulk of the papilla. Professor Ebner, of Vienna, in the "German Hand-book of Dentistry," 1891, by Scheff, Jr., claims that we have interrupted poorly-preserved specimens when we speak of medullary corpuscles as the dentine-formers. In view of this assertion, I will draw the gentleman's attention to those illustrations taken from perfect specimens, and ask him how he can account for the absence of odontoblasts, and in their stead, medullary tissues. Obviously there is a series of tissue-changes preceding the appearance of dentine, and one of the links in the chain is the odontoblast. At the lateral portion of the cuspid the question of the formation of enamel may possibly be settled.

Between the fully-developed enamel and the row of ameloblasts there is a broad layer of medullary tissue, considerably broader, indeed, than in any specimen of developing human teeth I have ever seen before. Ebner militates against the view "that the ameloblasts are not direct enamel-formers, but only transient formations, originating from a coalescence of medullary corpuscles, before the appearance of enamel-tissue." Can he or any one explain, may I ask, the composition of enamel-prisms of square or many-sided blocklets (admitted by all observers), except by the construction of each enamel-rod by a succession of medullary corpuscles, infiltrated with lime-salts?

It is unnecessary for me to state here that I seriously object to the assertion of Ebner, or any one else, that our specimens were or are imperfect, the greatest care having been taken in their preparation, and I can vouch for their perfection. With equal propriety I might ask the learned professor why it is that his specimens were so perfect as to not show the fibre between the enamel-prisms, nor the medullary tissue between the ameloblasts proper and the formed enamel.

The permanent cuspid has a papilla considerably broader than that of the temporary, so much so that it could be illustrated entirely only with a power of twenty-five diameters. The labial aspect is far more bulky than the lingual, the former being precipitous, the latter more tapering. It is composed of medullary, without the slightest admixture of myxo-fibrous or fibrous connective tissue, at the same time being poorly supplied with blood-vessels. Both the dentine- and enamel-caps are as yet narrow, terminating upon the labial side abruptly, an apparent stricture presenting at their termination, beyond which the papilla bulges considerably, while on the lingual side the dentine- and enamel-caps follow the unbroken line of the papilla.

The first temporary molar, at this stage of development, is of considerable size and importance. It presents in this section two cusps, of which the lingual is quite noticeably higher than the buccal, although the latter is but little less developed than the former. The papilla is a bulky mass of myxo-fibrous connective tissue, abundantly supplied with capillary blood-vessels. Corresponding to the valley between the two cusps, the papilla is narrowest, bulging from this point upward and outward. The summit of the papilla of the lingual cusp is higher and more pointed than that of the buccal. The papilla exhibits a row of odontoblasts, and only at the lower third of the lingual tissue, without a trace of odontoblasts. The summit of the papilla of the lingual cusp exhibits a zone of myxomatous tissue similar to that described in the papilla of the cuspid. The dentine-cap forms a continuous investment around the cusps, being narrow in the valley between them, and slanting towards the base of the papilla, reaching farther downward upon the buccal than upon the lingual side. It is fully developed, and composed of a narrow non-calcified and a broad calcified layer, the broader line between the two portions being more globular in the buccal than in the lingual cusp. The enamel is likewise continuous, fully developed, and calcified, being a trifle broader at the summit of the lingual than at that of the buccal cusp. In the valley between the cusps it appears somewhat broader than the layer of dentine.

The first permanent bicuspid, the product of the bud from the first temporary molar, at birth corresponds to a temporary tooth four and a half months old. It is cone-shaped and composed of medullary tissue, with but scanty capillary blood-vessels at the base of the cone. As a matter of course, there is not even a trace of odontoblasts visible. The papilla is covered with a layer of columnar epithelia, the so-called inner epithelium of the enamel-organ, not as yet transformed into ameloblasts.

The point of recurvation of the inner into the outer epithelium is deeper down upon the buccal than upon the lingual aspect of the papilla. The outer epithelium is still recognizable as being composed of short columnar epithelia, surrounded by fibrous connective tissue. Between the inner and the outer epithelium there is a well-developed myxomatous reticulum, *the enamel-organ.* The intermediate layer is present, though as yet little pronounced. In the specimen from which the figure is taken, the upper portion of the enamel-organ is torn and partly missing.

The second temporary molar has in our specimen two well-developed cusps, of which the lingual far surpasses in size the buccal. This will, as a matter of course, not show the relation between the two cusps with certainty, since it is quite possible that the lingual cusp was caught by the knife at its centre, therefore at its greatest height, while the buccal cusp may have been taken in a more peripheral portion, and thus appear smaller than it really is. Between the two cusps there is an elevation covered only by dentine, which likewise may represent a cusp cut near its periphery. The papilla is mainly myxo-fibrous connective tissue, and freely supplied with blood-vessels. Rows of odontoblasts are seen only at a small portion of its periphery. The height of the papilla is three times greater at the lingual than at the buccal portion. A striking feature is the sharp boundary-line at the lingual aspect, between the papilla, covered with dentine, and that without it, and the bulging out of the latter. The dentine-cap is present all over the upper surface of the papilla, somewhat broader at the summit of the lingual cusp than on the summit of the buccal. It is a trifle broader at the height of the central elevation than in the valleys either side of it.

The difference between the calcified and the non-calcified portions of the dentine is quite marked. There are two enamel-caps, formed one over each the lingual and the buccal cusp; the former being almost twice the breadth of the latter.

The second permanent bicuspid is still younger in its development; it corresponds to a temporary tooth in the fourth month of embryonal life. The papilla is a blunt cone divided into a conical upper portion, surrounded by the inner epithelium, and a broad base surrounded by fibrous connective tissue. The papilla is made up altogether of medullary tissue, and shows blood-vessels at its base in small numbers. The inner epithelium is quite conspicuous by the columna of its constituent elements. The same elements also produce the row of outer epithelia, which appear shortened only at the summit of the enamel-organ. The enamel-organ itself is not as yet fully developed, its points of intersection being large, the meshes, on the contrary, narrow. The intermediate layer is but little pronounced. In this specimen the enamel-organ was unbroken.

The first permanent molar is an unusually well developed tooth in our series. It has two well-pronounced cusps, the largest being the lingual. The papilla is composed mainly of myxomatous and medullary tissue, with an admixture of some delicate fibrous connective tissue. Its vascular supply is as yet scanty. It shows deep incisions on both the lingual and the buccal aspect, corresponding to the dentine-caps, beneath which the papilla bulges quite noticeably. Rows of odontoblasts are seen along the greater extent of the papilla; especially is this the case upon the lingual aspect, which shows an uninterrupted row of these formations. A second row is seen in the valley between the two cusps, where there is as yet no dentine formed. There are separate dentine-caps for each cusp, that over the lingual cusp being especially well developed.

The enamel-caps are, as is the rule in all developing teeth, shorter than the dentine-caps, that of the lingual cusp being at its summit twice as thick as that upon the buccal. The row of inner epithelia is transferred into ameloblasts over both cusps, and there is a distinct layer of medullary tissue present between the ameloblasts and the fully-developed enamel. The outer epithelium begins to break up at its upper portion, where the enamel-organ forms a broad layer of myxomatous tissue.

Huxley (*Quarterly Journal of Microscopical Science*, 1853), as early as 1853, says, when speaking of the development of dentine, "that it is not explicable by the cell theory." How true this statement, made so many years ago! We do not, however, agree with his assertion that the pulp-tissue takes no part in the formation of dentine. Any one considering the so-called cells as stable and unchangeable formations will be at a loss to explain the formation of any tissue of the teeth. The latest researches in histology have proved that as far as the morphological elements are concerned there is nothing stable during the advancing formation of the organism and its constituent parts, nor during full development, at the height of life; and far less is this the case during its decline. Before a tissue is fully formed there are repeated oscillations forward and backward in the appearance of morphological elements, the intervening stage being their reduction into the stage of indifferent medullary or embryonal tissue. It has been proved that the papilla of the developing teeth may proceed to the formation of myxomatous, nay, myxo-fibrous connective tissue. At its periphery this tissue, sprung from medullary corpuscles, falls back to medullary corpuscles, which unite into large branching protoplasmic bodies, resembling columnar epithelia, the so-called odontoblasts. These are by no means the dentine-formers proper, no more so than that osteoblasts are the real bone-formers. Both these formations are nothing but a pre-stage towards the formation of either dentinal or bone-tissue. Odontoblasts break up once more into medullary corpuscles, from which at last, by their infiltration with basis-substance and immediate deposition of lime-salt, they are first non-calcified, and at last calcified into dentine. The zigzag line of development of dentine, therefore, is first embryonal or medullary tissue; second, myomatous or myxo-fibrous tissue; third, embryonal or medullary tissue; fourth, odontoblasts; fifth, again embryonal or medullary tissue; and sixth and lastly, non-calcified and calcified dentine.

Every reduction to embryonal tissue is followed by a step in advance in the development of the organ, until at last the most perfect tissue, such as dentine, will make its appearance. It is a question in my mind whether the dentine as we see it at the time of birth is a lasting formation, or the same as we see it in the fully-grown temporary or permanent tooth. The size of the papilla and the dentine-cap are at birth far too small in comparison with what we see at the time of eruption. It is quite possible, therefore, that the first-formed dentine-cap is not lasting, and may eventually be reduced once more into medullary tissue, before the permanent breadth of dentine corresponding to the transverse diameter of a fully-formed tooth is reached. Similar oscillation must, of necessity, take place in the production of the pulp and the dentine of the roots. At the time of birth only the papilla of the crown is present, sharply bordering downward by fibrous connective tissue. This latter tissue is reduced to its embryonal condition in order to produce the necessary material for the production of the dentine of roots. Future observations will be required in order to settle the question as to how the dentine and cementum of the roots are formed.

The same puzzle we meet with in the history of development of the enamel. At the time of birth the myxomatous enamel-organ is far too small to enable us to understand the formation of a broad enamel-layer, such as we see at the time of eruption. Even the first established enamel-caps are far too small in comparison with the diameter of the crowns of either temporary or permanent teeth. Heitzmann and Bödecker, in their above-quoted publication, the result of eight years' hard labor, came to the conclusion that the elements of the original epithelial pegs are reduced to medullary tissue, afterwards to fibrous connective tissue, next to medullary tissue, and eventually to ameloblasts. Ameloblasts, according to their notion, are not direct enamel-formers, no more than odontoblasts are direct dentine-formers. The ameloblasts are reduced to medullary corpuscles, which after infiltration with the basis-substance, and immediate deposition of lime-salts, at last produce enamel-prisms.

From a study of their specimens and my own I am convinced that these views are correct.

Should the first appearing enamel-cap prove too small for a full-grown tooth, nothing is left to solve the puzzle of development but the assumption that even the fœtal enamel-cap is not lasting, and must undergo reduction or possibly repeated reductions to medullary tissue before it reaches the extent of a fully-developed tooth, such as we see at the time of eruption.

